# A lytic bacteriophage isolate reduced *Clostridium perfringens* induced lesions in necrotic enteritis challenged broilers

**DOI:** 10.3389/fvets.2022.1058115

**Published:** 2022-12-22

**Authors:** Chake Keerqin, Katherine McGlashan, Thi Thu Hao Van, Helene N. Chinivasagam, Robert J. Moore, Mingan Choct, Shu-Biao Wu

**Affiliations:** ^1^School of Environmental and Rural Science, University of New England, Armidale, NSW, Australia; ^2^EcoSciences Precinct, Department of Agriculture and Fisheries, Dutton Park, QLD, Australia; ^3^School of Science, RMIT University, Bundoora, VIC, Australia

**Keywords:** bacteriophage, *Clostridium perfringens*, necrotic enteritis, lession, broiler

## Abstract

**Background:**

Bacteriophages are viral predators of bacteria and are common in nature. Their host-specific infections against specific bacteria make them an attractive natural agent to control bacterial pathogens. Interest in the potential of bacteriophages as antibacterial agents in the production animal industries has increased.

**Methods:**

A total of 18 bacteriophages were isolated from Australian commercial poultry environments, from which three highly active phages were chosen for enrichment. Sequencing libraries were prepared using a Nextera XT kit (Illumina) and sequenced on an Illumina MiSeq instrument using 2 × 300 bp paired-end chemistry. The sequence data were then assembled and aligned with a2 bacteriophage as the reference. An animal trial was performed by oral gavaging *Clostridium perfringens* netB containing strain EHE-NE18 to the Ross 308 broiler chickens prior inoculation with *Eimeria* species. The chickens were raised following the management guide for Ross 308 from d 0 to d 21 and fed with starter and grower diets met the specific breed nutrient requirements. Body weight gain and feed intake were measured on d 9 and d 21 and FCR adjusted with mortality was calculated.

**Results:**

The isolated bacteriophages only had only 96.7% similarity to the most closely related, previously characterized, *Clostridium* bacteriophage indicated that they might represent a novel strain of bacteriophage. A “cocktail” containing the three bacteriophages was capable of lysing four known disease-inducing *C. perfringens* strains *in vitro*. Oral administration of the bacteriophages cocktail to broilers challenged with necrotic enteritis markedly alleviated intestinal necrotic lesions in the duodenum and jejunum on day 16 post-hatch. The phage treatment significantly reduced the lesion scores of birds challenged with NE (*P* < 0.01), and the lesion scores between birds treated with the bacteriophages and the unchallenged birds were not statistically different (*P* > 0.05). However, no effect on the growth performance was observed during the recorded period of days 9-21.

**Conclusion:**

These findings suggest that bacteriophage treatment is a promising approach to protect intestinal health from *C. perfringens* induced necrotic enteritis. Further research will be required on the dosing, route of administration, and large scale validation studies to further advance this approach to pathogen control.

## Introduction

Necrotic enteritis (NE) is a bacterial enteric disease in poultry accountable for over US$6 billion profit loss annually to the industry worldwide in the treatments and containment measures (1). The etiology of NE has been well characterized and it is mainly due to the rapid proliferation of *C. perfringens* Type G (2) strains that express the NetB toxin (3), albeit NetB negative strains have also been reported to cause NE (4). Outbreaks of NE also depend on a number of predisposing factors that result in degradation of intestinal health and compromise of the host immune status (3, 5, 6). It is reported that change of feed, use of different cereal bases in diet formulation, i.e., non-viscous grains to viscous grains, the inclusion of low digestibility protein sources, *Eimeria* infection, and compromise of to the host immune status, all exacerbate the chances of NE outbreaks (7). Flocks affected by acute NE exhibit clinical symptoms coupled with a rapid increase in flock mortality and distress in the affected bird populations, but subclinical (mild) disease are more frequent with little mortality rate if any, and suboptimal growth performance. The subclinical form of necrotic enteritis leads to more significant economic consequences with prolonged deterioration of growth performance resulting from loss of feed efficiency over time (8).

For decades, poultry and other animal production systems across the globe have depended on in-feed antibiotics to sustain growth performance and control enteric pathogens as they can effectively maintain intestinal microbiota uniformity and suppress bacterial infection (9). However, the emergence of antibiotic-resistant bacteria and the possibility of horizontal transfer of antibiotic resistance genes to human pathogens resulted in the ban of in-feed antibiotics and has ushered in antibiotic-free practices that require alternatives to conventional in-feed antibiotics to maintain flock performance and health. The suppression of specific intestinal bacterial populations is essential to manage opportunistic pathogens and stop them from reaching a disease-causing threshold (10). Following the 2006 EU's legislative ban on antibiotic use in animal feed, numerous countries across the globe have followed suit and are dealing with the challenges of running antibiotic-free production systems. Early efforts to overcome these challenges focused on the adoption of antibiotic-free measures that provided some protection against pathogens (11), but the end results have been inconsistent.

The lack of consistency in the efficacy of the current alternatives for maintaining performance means that novel solutions continue to be explored. The interest in bacteriophages stems from the fact that they naturally target specific bacteria and have narrow host ranges. On exposure to specific bacteriophages, a host bacterium undergoes lysis resulting in the death of the bacterium and the release of bacteriophages that can continue to infect other host bacteria; this self-replicating process is a very different process compared to other alternatives to antibiotics. Hence, bacteriophages are an attractive and feasible option for pathogen control. The use of phage therapy is not new. In fact, the clinical application of bacteriophages was first implemented in the early 1920s, with extensive therapeutic application in human medicine across the eastern European countries, and it has continued to the present time (12). Many known attributes of bacteriophages fit the criteria for an effective antibacterial alternative, including automatic dosing capability through lytic propagation of bacteriophages progeny, minimal inherent toxicity, low disruption to commensal microbiota, biofilm clearing, and variable routes of administration (13). Bacteriophage absorption on the surface of susceptible bacteria is mediated by specific receptors on the bacteria cell wall (14). Different types of bacteriophages that infect any one species of bacteria may recognize different receptors. Hence, the combination of several bacteriophages in the form of a cocktail may improve their efficacy against a target bacteria. Indeed, the use of multivalent bacteriophage cocktails was reported to be successful in reducing mortality and improving performance in chickens infected with *C. perfringens* resulting in clinical NE (15).

However, only limited research has been reported on phage prophylactic treatment against *C. perfringens* induced subclinical necrotic enteritis in chickens. This study was conducted to isolate and purify bacteriophages from poultry production environments that could infect and lyse *C. perfringens*, determine their lytic profile against a range of known virulent *C. perfringens* strains capable of inducing NE, and then the isolated bacteriophage was evaluated for prophylactic properties *in vivo* using a necrotic enteritis challenge model.

## Materials and methods

### Sample collection, bacterial hosts, and bacteriophage preparation

Samples from various sources within poultry environments were aseptically collected for bacteriophage isolation, which included soil samples from backyard chicken sheds and yards located in Armidale and Tamworth, NSW, Australia, sewage and offal wash from seven individual chicken farms, and intestinal content of chickens from experiments conducted at UNE (Armidale, New South Wales, Australia).

All collected samples were kept on ice until processed. *C. perfringens* were isolated by plating serial dilutions in 0.1% peptone onto Perfringens Agar Base (Oxoid, Hampshire, UK) supplemented with Tryptose Sulphite Cycloserine (TSC) (Oxoid, Hampshire, UK), followed by incubation under anaerobic conditions using an anaerobic sachet (Oxoid Australia) at 39^o^C for 48 h. Putative *C. perfringens* colonies were re-streaked twice (to ensure purity) on fresh Horse Blood Agar (HBA) (Oxoid, Hampshire, UK) and incubated at 39^o^C for 18 h. Bacteria isolates were picked and suspended in the Brain Heart Infusion (BHI, Oxoid, Hampshire, UK) broth with sterile glycerol addition to 40% (v/v) and stored at −20^o^C until used.

Samples for bacteriophage isolation were prepared by gently shaking 1 g of solid samples (i.e., soil, gut content or litter) or 1 ml liquid sample (effluent) in 9 ml of SM buffer (0.1 M NaCl, 1 mM MgSO_4_, 0.2 M Tris-HCl, pH 7.5) for 12 h. The supernatant was then centrifuged at 11,000 × *g* at 4^o^C for 5 mins. The supernatant was then filtered through 0.22-μm-pore-size Millipore filters (MF-Millipore™, MERCK, Australia). Bacteriophages were isolated as per the methods of Smith (16) using the isolated *C. perfringens* host strains. In brief, overnight growth of *C. perfringens* from HBA agar was initially mixed in 3 ml brain-heart infusion (BHI) broth, and then further BHI was added to adjust the turbidity to 220 ± 15 Nephelometric Turbidity Unit (NTU). Serial dilutions of bacteriophage filtrates were prepared in SM buffer and 100 μl of each bacteriophage dilution was mixed with 200 μl of *C. perfringens* and incubated at 37^o^C for 30 min. Each set of the bacteriophages-host mixture was mixed into 7 ml of 0.5% BHI soft agar (supplemented with 1 mM MgCl_2_ and 1 mM Ca Cl_2_) and plated as an overlay on 1% BHI base agar. Plates were incubated anaerobically at 39^o^C for 48 h. Well separated plaques were picked and resuspended in 1 ml of SM buffer, then stored at 4°C overnight. Serial dilutions of the homogenized plaques were made and cultured with the susceptible host in three cycles of plaque purification (16, 17).

Phage stocks were prepared from three to five plates with confluent plaques from the third round of phage purification. SM buffer (5 ml) was added to confluent plates and shaken slowly at 10 rpm for 12 h to resuspend the bacteriophage. The supernatants were then filtrated through 0.22-μm-pore-size Millipore filters (MF-Millipore™, MERCK, Australia). The resulting filtrates were centrifuged at 40,000 × g at 4°C for 2h. The resulting bacteriophage pellets were resuspended in SM buffer to form 1 × 10^9^ pfu/ml bacteriophage stock and stored in the dark at 4°C.

### Lytic profile of the bacteriophage

The bacteriophages were tested for lytic ability against four pathogenic *C. perfringens* strains (NE14, NE18, NE21, and NE36) to investigate the bacteriophage's virulence against target hosts *in vitro*. The characteristics of the tested *C. perfringens* strains are detailed in Table 1. Bacterial lawns were prepared using turbidity-adjusted *C. perfringens* strains (220 ± 15 NTU) mixed with 7 ml of 0.5% BHI soft agar (supplemented with 5mM MgSO_4_ and 10mM CaCl_2_) and set on the prepared 1% BHI base agar. The bacteriophage lysates were titered, and all were within the range of 1–7 × 10^9^pfu/ml. A volume of 20 μl of each bacteriophage preparation was aseptically placed in duplicate on marked positions of the bacterial lawns and set to dry with minimal disturbance to retain the bacteriophage droplets at the designated positions. Plates were incubated anaerobically at 39°C up to 48 h and inspected for plaque formation. All positive tests were replicated three times with the relevant *C. perfringens* host.

**Table 1:** Characteristics of *C. perfringens* strains and susceptibility to the bacteriophages.

***C. perfringens*** ** isolate**	**Source of** ** location**	**Year** ** Isolated**	***netB gene***	**NE induction^1^**	**References**	**Phage** ** Susceptibility**
NE1	Australia	2002	–	No	(18)	–
NE4	Australia	2002	+	Unknown	(18)	–
NE7	Australia	2002	+	Unknown	(18)	–
NE14	Australia	2002	+	Unknown	(18)	+
NE16	Australia	2002	+	Unknown	(18)	–
NE18	Australia	2002	+	Yes	(18)	+
NE21	Australia	2002	+	Unknown	(19)	+
NE31	Australia	2004	+	Yes	(5)	–
NE36	Australia	2010	+	Yes	(20)	+
NE38	Australia	2011	+	Unknown	Lab stock	–

All strains were isolated from necrotic enteritis-affected chickens.

^1^NE induction in experimentally induced NE model.

### DNA sequencing of the bacteriophage

The bacteriophage stocks were concentrated by precipitation with polyethylene glycol, and DNA was isolated using a phenol/chloroform extraction method. Sequencing libraries of 5 bacteriophages from different sources (A2, C2, D1, E1, and H1) were prepared using a Nextera XT kit (Illumina). The libraries were sequenced on an Illumina MiSeq instrument using 2 × 300 bp paired-end chemistry. The sequence data generated from the MiSeq were assembled using the A5-miseq assembly pipeline (21). The bacteriophage DNA sequences were aligned using Clone Manager Suite V8 (Scientific and Educational Software, NC) with a2 bacteriophage as the reference. Homology analysis of the bacteriophage a2 sequence was performed against the NCBI database using the BLASTn program. Gene prediction was performed by submitting the DNA sequence of bacteriophage a2 to the RAST server (https://rast.nmpdr.org/) for RAST annotations. The whole genome sequence data has been deposited in the NCBI database with the accesion numbers OP753449–OP753453.

### Phage inoculants and *in vivo* study

Eighteen bacteriophage isolates that had lytic activity against the *C. perfringens* EHE-NE18 strain were enriched for the preparation of a bacteriophage inoculate. Three selected bacteriophages were prepared in larger volumes using the procedure adopted for purification as described above. Pelleted bacteriophages were resuspended in SM buffer to a final concentration of 1 × 10^6^ pfu/ml for the *in vivo* experiment.

Day-old birds (Ross 308 broilers) were obtained from a commercial hatchery (Baiada Hatchery, Tamworth, NSW, Australia). Birds were housed in 18 pens, each containing 11 birds. The pens (0.9 × 0.95 m) were equipped with feeders and drinkers and hardwood shavings were used as bedding materials. The temperature and lighting were controlled according to the Ross 308 broiler guideline. A standard wheat-soybean starter diet formulated to meet the 2014 Ross 308 nutrient specifications was fed to all birds from day 0 to 10 (Table 2). A grower diet consisting of the wheat-based formulation was fed from day 10 to the end of the trial on day 21 (Table 2). Feed and water were available *ad libitum*. Pen weight and feed intake were measured on days 9 and 21, and the feed conversion ratio (FCR) was calculated by pen feed intake divided by pen bird weight gain and adjusted with mortality during the rearing period of d 9 to d 21.

**Table 2:** Composition and nutrients of starter and grower.

**Ingredients (%)**	**Starter**	**Grower**
Wheat	37.8	36.6
Sorghum	20.0	26.7
SBM	28.3	20.6
Canola meal	3.50	5.40
Meat and bone meal	3.70	4.10
Canola oil solvent	3.42	3.92
Limestone	0.87	0.72
Dical Phos	1.07	0.77
Salt	0.10	0.10
Na bicarb	0.20	0.20
UNE Vit Pre	0.07	0.07
UNE TM	0.09	0.09
Choline	0.04	0.05
L-lysine HCl	0.40	0.36
DL-methionine	0.29	0.24
L-threonine	0.18	0.15
**Nutrient (% unless indicated)**		
ME Poultry (kcal/kg)	3,000	3,100
Crude Protein	23.0	20.8
Crude fat	5.68	6.49
Crude Fiber	3.23	3.21
Isoleucine	0.99	0.87
d Arg pou	1.34	1.16
d Lys pou	1.27	1.10
d Met pou	0.59	0.52
d M+C pou	0.94	0.84
d Trp pou	0.24	0.20
d Thr pou	0.83	0.73
d Val pou	0.94	0.84
NSP insolg/kg	7.62	10.17
Calcium	1.00	0.90
Available phosphorus	0.50	0.45
Sodium	0.16	0.16
Potassium	0.95	0.82
Chloride	0.21	0.21
Magnesium	0.22	0.21
Selenium mg/kg	0.72	0.72
Zinc mg/kg	242	243
Iron mg/kg	102	104
Copper mg/kg	38.6	38.6
Manganese mg/kg	219	220
Choline mg/kg	1600	1500
Vitamin AIU	16800	16800
Vitamin EIU	113	113
Vitamin K mg/kg	4.2	4.2
Thiamin mg/kg	7.6	7.6
Riboflavin mg/kg	13.1	13.0
Pantothenic acid mg/kg	51.4	58.6
Pyridoxine mg/kg	10.8	10.8
Biotin mg/kg	0.48	0.47
Linoleic 18:2	1.73	1.94

The experiment consisted of a control untreated and unchallenged group, and necrotic enteritis challenge groups with or without bacteriophage treatment. On day 9, the NE challenge groups were given 1 ml of a mixture of field strains of *Eimeria* species containing 5,000 oocysts each of *E. acervulina* and *E. maxima*, and 2,500 oocysts of *E. brunetti* (Eimeria Pty Ltd, Werribee, VIC, Australia) by oral gavage, and unchallenged control birds were orally gavaged with 1 ml phosphate buffered saline as a sham treatment. On days 14-15, birds in the challenged groups were inoculated by oral gavage with 1 × 10^8^ CFU of *C. perfringens* EHE-NE18 strain as described by Wu et al. (22). The bacteriophage treatment birds were orally gavaged with 1 × 10^6^ units of the *C. perfringens* specific bacteriophage inoculate approximately 1 h following *C. perfringens* gavage. On day 16, two birds per pen from all the groups were randomly selected, weighed, and euthanized by cervical dislocation to perform post-mortem analysis and intestinal lesion scoring at a 0–6 scale according to Keyburn et al. (19). On day 21, the experiment was completed, and birds were euthanized and disposed of according to the approved ethics protocol.

### Statistical analysis

Data were analyzed by one-way ANOVA with the pen as the experimental unit. Paired comparisons of means were performed using Tukey's test when differences among treatments were detected by ANOVA. Significance at *P* < 0.05 was declared for paired comparison.

## Results and discussion

### Lytic properties of *C. perfringens* specific bacteriophages and genomic characterization

**Figure 1:**
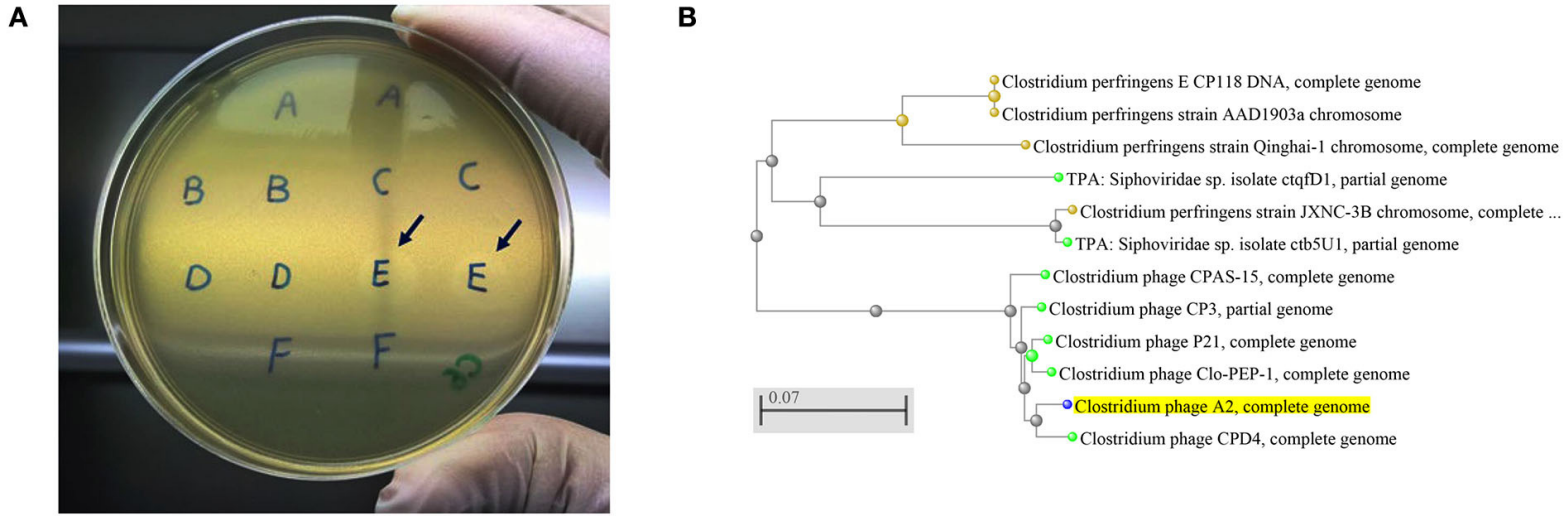
**(A)** *Clostridium perfringens* bacteriophage plaques. Arrows showing the plaques produced by the strains from sample E that was collected from chicken caecal content, **(B)** An evolutionary tree of *Clostridium* bacteriophage strains produced by NCBI online blast tools with the Fast Minimum Evolution tree method. The *Clostridium* phage A2 isolated in the current study is highlighted in yellow showing its close relation with *Clostridium* phage CPD4. The scale bar corresponds to 0.07 substitutions per nucleotide position.

The current study isolated and purified eighteen *C. perfringens* bacteriophages isolates. Each bacteriophage isolate was capable of producing plaques (Figure 1A) on each of the *C. perfringens* hosts obtained from the same poultry environments (soil, sewage, offal wash or intestinal content) and corresponding geographic locations. Among 46 field *C. perfringens* isolates from the environment, only three *C. perfringens* isolates were susceptible to all the bacteriophages isolates and thus used as the hosts for the bacteriophages. Therefore, all 18 bacteriophages were subsequently enriched with these three common hosts for bacteriophage purification, and the resulting bacteriophage preparations were used to perform lytic tests individually against pathogenic *C. perfringens* strains derived from chickens with necrotic enteritis. Positive lytic activities were observed for all these bacteriophage strains against four previously described virulent *C. perfringens* strains (NE14, NE18, NE21, and NE36) in our spot-testing assay. However, other strains of *C. perfringens* (NE1, NE4, NE7, NE16, NE31, and NE38) were not susceptible to these bacteriophages (Table 1). As the bacteriophage preparation was lytic against NE18 strain, which has been reported to produce NetB toxin (3) and has been used in NE experimental disease induction models (23), this preparation was used as an inoculate in the subsequent *in vivo* experiment with EHE-NE18 as the challenge strain.

A narrow spectrum of infectiousness against *C. perfringens* strains also presents the possibility of a controlled elimination of pathogenic *C. perfringens* strains but minimal harm to commensal *C. perfringens*. It has been demonstrated that *C. perfringens* exists as normal gut commensals with low abundance in the intestinal tract of healthy chickens (6).

Five bacteriophages genomes were randomly chosen for sequencing, and the BLAST analysis against the NCBI database indicated the bacteriophages isolated aligned with the *Clostridium* bacteriophages CPD4 with a homology of 97.7% which clustered closely as shown in Figure 1B and 77 genes were predicted, of which 19 had specific bacteriophage functions. The degree of homology to previously characterized bacteriophages indicates that the bacteriophages isolated in this study might represent a novel bacteriophage strain. *C. perfringens* specific bacteriophage isolates have been reported to belong to the family *Siphoviridae* or *Podviridare*, and bacteriophages endolysin has been shown to be responsible for the lytic activity (17, 24). The sequences of the bacteriophage isolates produced in the current study generated a single contig with the same GC content (34%) and showed a similar genome size with 99.98–100.00% identities (Table 3). The genomes of several *C. perfringens* specific bacteriophages have been characterized as reported previously, providing essential information for understanding the virulence of phages to their hosts and for downstream applications (17, 25).

**Table 3:** A5-miseq assemblies of the bacteriophages isolates.

**Strains**	**Contigs**	**Genome Size**	**% GC**	**Match** ** (%)**
Phage a2	1	52794	34	–
Phage c2	1	52795	34	99.99
Phage d1	1	52794	34	99.99
Phage e1	1	52792	34	99.98
Phage h1	1	52794	34	100.00

### Bacteriophage treatment reduced NE induced lesions

No mortality of birds due to NE and low lesion scores (0.8 out of a maximum of 6) were observed in the *in vivo* challenge experiment, indicating that subclinical NE had been induced. The lesion score results are shown in Table 4. Higher lesion scores were recorded in both the duodenum (*P* < 0.01) and jejunum (*P* < 0.01) of the challenged birds than those unchallenged. The phage treatment significantly reduced the lesion scores of birds challenged with NE (*P* < 0.01), such that the lesion scores between birds treated with the bacteriophages and the control unchallenged birds were not statistically different (*P* > 0.05). However, this significant effect of the bacteriophages on lesion scores did not fully manifest in the measures of bird performance. In the current study, the birds under NE challenge were significantly affected, showing reduced BWG, FI, and increased FCR in the challenged treatments compared to the control (Table 5). NE challenged birds with or without bacteriophage treatment had reduced BWG and FI and increased FCR during days 9 to 21 or 0 to 21 (*P* < 0.001 for all except FCR during d 0–21 being *P* = 0.002). The challenge model used in the current study experimentally reproduced the conditions of possible NE outbreaks in the field, of which coccidiosis serves as a predisposing factor to damage intestinal epithelium before *C. perfringens* infection (7, 22). The current study was not set out to apportion the impact of the *Eimeria per se*, and therefore, it is difficult to determine with any certainty to what extent the lack of performance improvement by the bacteriophage therapy could be due to its inability to counter any damage that resulted from coccidiosis. *Eimeria* negatively affects nutrient utilization by interfering with the integrity of epithelium cells, leading to compromised growth of the animals (6, 26).

**Table 4:** Lesion scores of the birds under treatments at day 16^*^.

**Treatment**	**Duodenum**		**Jejunum**		**Ileum**	

	**Mean**	**SE**	**Mean**	**SE**	**Mean**	**SE**
Control	0.0^b^	0.0	0.0^b^	0.0	0.0	0.0
NE only	0.8^a^	0.1	0.7^a^	0.1	0.6	0.3
NE+ bacteriophages	0.2^b^	0.2	0.1^b^	0.1	0.3	0.2
*P*-values	0.005		0.005		0.095	

* Means within the same column with same letters are not significantly different; SE: standard error.

**Table 5:** Performance of the birds in response to the NE and bacteriophages treatments^*^.

**Treatment**	**Control**		**NE only**		**NE**+ **bacteriophages**		***P*-values**

	**Mean**	**SE**	**Mean**	**SE**	**Mean**	**SE**	
**Day 0–9**							
BWG	239	3	244	2	243	1	0.308
FI	206	2	210	3	208	2	0.471
FCR	1.021	0.029	1.015	0.031	1.011	0.013	0.797
**Day 9–21**							
BWG	748^a^	18	500^b^	12	516^b^	20	0.000
FI	927^a^	22	757^b^	14	775^b^	31	0.000
FCR	1.238^b^	0.010	1.516^a^	0.082	1.511^a^	0.180	0.001
**Day 0–21**							
BWG	950^a^	21	707^b^	13	722^b^	20	0.000
FI	1128^a^	22	957^b^	14	974^b^	30	0.000
FCR	1.188^b^	0.013	1.355^a^	0.056	1.353^a^	0.118	0.002

* Means within the same row with same letters are not significantly different; SE, standard error.

Although bacteriophage treatment did not translate into improved bird performance, less severity of intestinal lesions in the treated birds suggests that bacteriophages appropriately selected against the NetB producing strain of *C. perfringens* may be worthy of further exploration.

The current study focused on the performance of the birds during days 9–21, when the NE challenge was applied, and the NE effect was expected. However, a prolonged rearing period might be useful to observe the bacteriophage treatment effect on later stage performance through improved intestinal health. In addition, the dosage and the route of bacteriophage administration may also be important factors for the effectiveness of the bacteriophage treatment to protect birds from NE infection. These will need to be addressed in future studies to maximize the protective effect of bacteriophages, such as water and feed delivery and higher doses. Further, more bacteriophage strains may be needed to control *C. perfringens* strains in chickens through the administration of a wider spectrum cocktail.

In conclusion, 18 isolated *C. perfringens* specific bacteriophage strains were isolated. The finding that they had 96.7% similarity to the most closely related, previously characterized, *Clostridium* bacteriophage indicated that they represent a similar cluster of *Clostridium* bacteriophage. The result showed an encouraging protective effect of the bacteriophage application with alleviated lesions in the gut of NE-challenged birds despite the fact that no performance improvement was seen. Whether bacteriophage can be used in the poultry production to combat NE is yet to be assured. Further investigations are warranted to optimize the dosage and administration protocol and to isolate more NetB positive and even negative bacteriophage strains for a wider spectrum control of different *C. perfringens* pathogens.

## Data availability statement

The data presented in the study are deposited in the NCBI GenBank repository, accession numbers OP753449 – OP753453.

## Ethics statement

The animal study was reviewed and approved by the Animal Ethics Committee of the University of New England (UNE), Armidale, Australia.

## Author contributions

CK performed data collection and analysis, interpreted results, and wrote the manuscript. KM performed isolation of the bacteriophages and analysis. TV sequenced the bacteriophage genome, analyzed data, and read the manuscript critically. HC participated the design of the project, isolated bacteriophages, and reviewed the manuscript critically. RM participated the design of the project, data analysis, and reviewed the manuscript critically. MC participated the conceptualization of the project and reviewed the manuscript critically. S-BW conceptualized, designed the project, performed the animal experiment, analyzed data, and reviewed the manuscript critically. All authors contributed to the article and approved the submitted version.
